# Mass Transfer Coefficient in Multi-Stage Reformer/Membrane Modules for Hydrogen Production

**DOI:** 10.3390/membranes8040109

**Published:** 2018-11-14

**Authors:** Diego Barba, Mauro Capocelli, Marcello De Falco, Giovanni Franchi, Vincenzo Piemonte

**Affiliations:** 1 Unit of Process Engineering, Department of Engineering, Università Campus Bio-Medico di Roma, via Álvaro del Portillo 21, 00128 Rome, Italy; diego.barba@unicampus.it (D.B.); m.defalco@unicampus.it (M.D.F.); franchi.giovanni@outlook.it (G.F.); 2 Unit of Chemical-physics Fundamentals in Chemical Engineering, Department of Engineering, Università Campus Bio-Medico di Roma, via Álvaro del Portillo 21, 00128 Rome, Italy; v.piemonte@unicampus.it

**Keywords:** physical-mathematical modelling, concentration polarization, steam reforming, palladium membranes, experimental data

## Abstract

Hydrogen is a promising energy carrier, and is exploitable to extract energy from fossil fuels, biomasses, and intermittent renewable energy sources and its generation from fossil fuels, with CO_2_ separation at the source being one of the most promising pathways for fossil fuels’ utilization. This work focuses on a particular configuration called the Reformer and Membrane Module (RMM), which alternates between stages of Steam Reforming (SR) reactions with H_2_ separation stages to overcome the thermodynamic limit of the conventional SR. The configuration has numerous advantages with respect to the more widely studied and tested membrane reactors, and has been tested during a pilot-scale research project. Although numerous modelling works appeared in the literature, the design features of the material exchanger (in the so-called RMM architecture) of different geometrical configurations have not been developed, and the mass transfer correlations, capable of providing design tools useful for such membrane modules, are not available. The purpose of this work is therefore to apply a physical-mathematical model of the mass transfer, in three different geometries, considering both concentration polarization and membrane permeation, in order to: (i) simulate the cited experimental results; (ii) estimate the scaling-up correlations for the “material exchange modules”; and (iii) identify the mass transfer limiting regime in relation to the gas mass flow rate.

## 1. Introduction

Hydrogen is a promising energy carrier, and is exploitable to extract energy from fossil fuels, biomasses, and intermittent renewable energy sources [1,2]. Generating hydrogen from fossil fuels is one of the most promising alternatives in the framework of carbonaceous fuels’ utilization, with simultaneous CO_2_ sequestration [1,2]. The adoption of H_2_ as an energy vector would represent a radical change in the energy sector, impacting its production, distribution, and consumption since it can be converted into both electrical power and heat using fuel cells or combustion engines without generating CO_2_ emissions locally [3,4,5].

The current world hydrogen consumption is more than 50 Mton/year [1] and is consistently being devoted to the chemical and petrochemical sectors, such as for ammonia and methanol synthesis, hydrogenation, hydrocracking, and hydrodesulphurization processes, with a very small fraction currently being used for energy purposes [1,2,3,4].

Nowadays, the most reliable and cheapest way to produce H_2_ is the steam reforming (SR) of light hydrocarbons (natural gas, gasoline) [1,2] which costs in the range of 2–4 dollars/kgH_2_ [1]. The technology for SMR is well-developed and applicable to a wide range of scales, from 1 t/h H_2_ (small decentralized units) to about 100 t/h (large ammonia manufacturing plants), and renewable energy sources will hardly replace these sources in the near future. At present, the global warming potential (GWP) of hydrogen production via the SMR process is around 10 kg CO_2_ /kg of H_2_ produced [4,6,7,8,9].

Even including the costs of CO_2_ recovery and sequestration, the cost of hydrogen production from fossil fuels is expected to be much lower than alternative production routes (e.g., electrolysis) in large-scale markets [8,9,10]. Looking at the projected trends (150 million tonnes by 2040), it may replace more than 18 million barrels/day of petroleum, assuming that the hydrogen fuel cell vehicles have been made 2.5 times more efficient than gasoline cars by that time [11,12].

The Membrane Reactors (MR) are manufactured by including selective membranes directly inside the reaction environment (e.g., in the catalytic tubes) so that the hydrogen produced is immediately removed [13]. The MR configuration has been extensively tested and discussed as the solution to overcome the thermodynamic limit of SR, thanks to the continuous separation of the produced H_2_ [3,4,5,6,13,14,15]. Pd-based supported membranes are the most promising type, thanks to the very high selectivity and good permeation flux, resulting in H_2_ purities of >99%. Besides the costs of precious metals, the major challenges for their complete affirmation are the mechanical stability of thin membranes, as well as the chemical stability (e.g., poisoning by CO and H_2_S) [4,5,6]. Some interesting results appeared also in the context of dense ceramic and microporous membranes, although there is not a clear winner from a commercial point of view [4,5,6].

**Figure 1:**
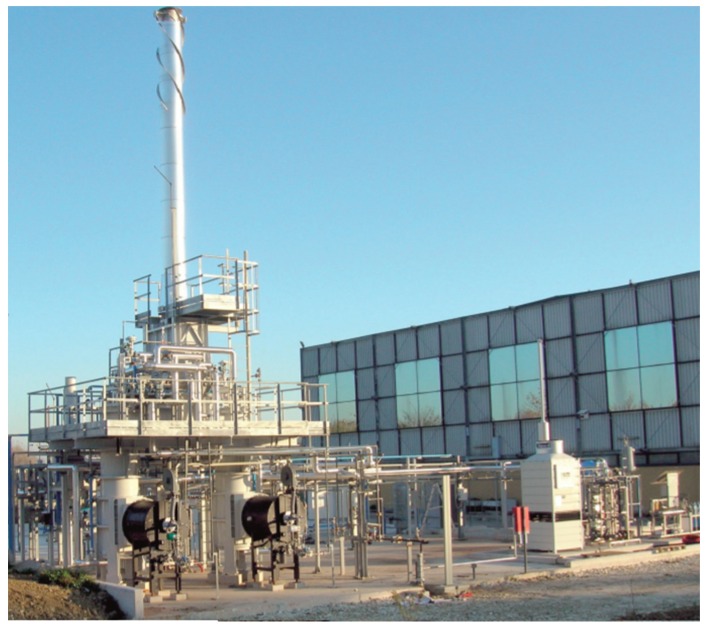
Overall view of the Pilot Plant [21].

An alternative approach is to arrange the separation modules downstream to reaction units, creating multi-stage reactor-membrane modules (RMM configuration), which is the object of this study. An innovative 20 Nm^3^/h prototypal RMM plant has been developed, designed, built, and tested during the Research Project (founded by MIUR, Italy), entitled “Pure hydrogen from natural gas reforming up to total conversion obtained by integrating chemical reaction and membrane separation” [16]. The pilot plant is represented in Figure 1 and comprises of two-step reformers and membrane modules working at 550–650 °C. It was built and tested by Tecnimont-KT Kinetics Technologies in Chieti (Italy) and represents the unique example of this technology at a Technology Readiness Level higher than 6 [17,18]. More than 1500 h of experiments on three types of commercial membranes addressed the potential of selective membrane application in industrial high-temperature chemical processes. The RMM, based on two stages of reaction and separation, allows an exceedance of equilibrium conversion of about 20%. The higher the removal of hydrogen carried out with the membrane, the higher the increase of global feed conversion. By increasing hydrogen recovery of up to 50% and 70% through the sweeping steam, the two-stage RMM configuration may allow an exceedance of the equilibrium conversion of up to 30 and 40%, respectively. RMM architecture working at 600 °C and 650 °C may reach a conversion of up to 72 and 90%, respectively, with four stages [16,17,18,19,20]. Currently, Reinertsen and SINTEF are working on a similar project with the aim of realizing a package unit of a pilot 40 ft container producing 25–100 Nm^3^/h of hydrogen [21].

The first experimental data collected at the Chieti Pilot plant during the testing phase, as well as the average H_2_ permeability at the operating conditions, can be found in our previous works [16,17,18,19]. The authors have also assessed and compared the benefits and drawbacks of the MMR configuration in relation to the MR [17,18,19,20,22]. Globally, the Research Project proved that the RMM presents a production cost which is 10% lower than those of the conventional H_2_ scheme, allows for implementation of a direct CO_2_ sequestration unit [22], and shows the following main advantages with respect to the MR arrangement:

- The MR is mechanically complex and presents a large and unpractical heat transfer surface—in the MR, the concentric tube geometry yields an imbalance between the surfaces required for heat transfer (outer tube) and the available surface for mass transfer (of the inner membrane tube);
- RMM enables the de-coupling of separation and reaction operating temperatures, increasing the stability and the durability of the membranes and enabling independent optimization of the reforming temperature;
- RMM simplifies the mechanical design of membrane tubes compared with the one embedded in a catalyst tube, and a simple shell and tube geometry can be selected for the tubular separation module;
- RMM simplifies maintenance of the Pd/Ag membrane modules and catalyst replacement.

With these premises, we analyzed multiple data related to the pilot plant experiments, and through the mathematical modeling dedicated to the membrane modules, we also estimated the mass transfer coefficients. By selecting several data at different flow-rates, gas compositions, temperatures, and different geometrical configurations, we calculated semi-empirical correlations useful for scaling up the RMM. The aforementioned mathematical model has been carefully validated through experimental data in order to extrapolate the values of the transport coefficients and the correlations necessary to scale-up the “material exchangers”, which represents the core of the RMM architecture.

From a permeation modelling point of view, a first benchmark in the theoretical modelling of H_2_ permeation trough a Pd self-supported membrane has been developed by Ward et al. [23]. The model was utilized to individuate the rate-limiting processes among the fundamental kinetic steps: (i) external mass transfer (binary mixtures); (ii) surface adsorption and desorption; (iii) transitions to and from the bulk metal; and (iv) diffusion within the metal. On this basis, Caravella et al. modelled the transport in Pd-alloy membranes, considering both the non-ideal hydrogen transportation in membrane (influences of support, inhibition by CO by the Sievert-Langmuir equation, and the effect of membrane polarization) and external mass transfer resistance on hydrogen permeation [24,25,26]. Their results are reported as an “adjusted” pressure exponent of a Sieverts-type empirical law, and it is function of several factors, such as temperature, total pressure, membrane thickness, and non-ideal behaviours. The theoretical meaning of the adjusted-Sieverts exponent can be explained by dividing the overall hydrogen permeation into several elementary steps (adsorption, desorption, diffusion in the Pd-based layer, and the two transitions, surface-to-bulk and bulk-to surface) [26,27]. Sarti et al. tested a Pd_80_-Ag_20_ (NGK) in a shell and tube configuration with different mixtures and operating conditions. The experimental tests were performed with a mixture of H_2_/N_2_/CH_4_. At the same time, a theoretical model based on the previous model of Ward et al. was developed to analyze the competitive adsorption of hydrogen and carbon monoxide molecules. The experimental tests show the existence of a concentration polarization phenomena due to non-permeable species [28,29,30,31]. In the first experimental setup [28], the Sherwood number followed a boundary-layer type of correlation, whereas in the second [30], a linear correlation between the Sherwood and Péclet numbers was found. Globally, the concentration polarization has been extensively discussed in the literature, and has been included in several models and at different conditions, mainly by modifying the exponent for the pressure dependence in the Sievert law equation [26,27,28,29,30,31,32].

Other authors discussed the transport-reaction-permeation regimes, also addressing the competitive adsorption limiting the overall H_2_ permeation [14,15]. Barbieri et al. [33] interpreted the observed decrease in hydrogen flux through a palladium-silver membrane over time with a CO inhibition (by up to 2 bars) in terms of a Sieverts-Langmuir model, assuming a linear correlation between the decrease in hydrogen permeance and surface coverage by carbon monoxide. Consequently, they accounted for the membrane surface fraction not available for hydrogen permeation using a Langmuir affinity constant for carbon monoxide and a temperature-dependent “permeance reduction factor” [33].

To the best of our knowledge, although numerous modelling works have appeared in the literature, the design features of the material exchanger (in the so-called RMM architecture) of different geometrical configurations have not been developed, and the mass transfer correlations, capable of providing design tools useful for such modules, are also not available.

The purpose of this work is therefore to apply a physical-mathematical mass-transfer model at three different geometries, and by considering both concentration polarization and membrane permeation, to simulate the cited experimental results, thereby estimating the scaling-up correlations for the “material exchange modules”.

## 2. Materials and Methods

**Figure 2:**
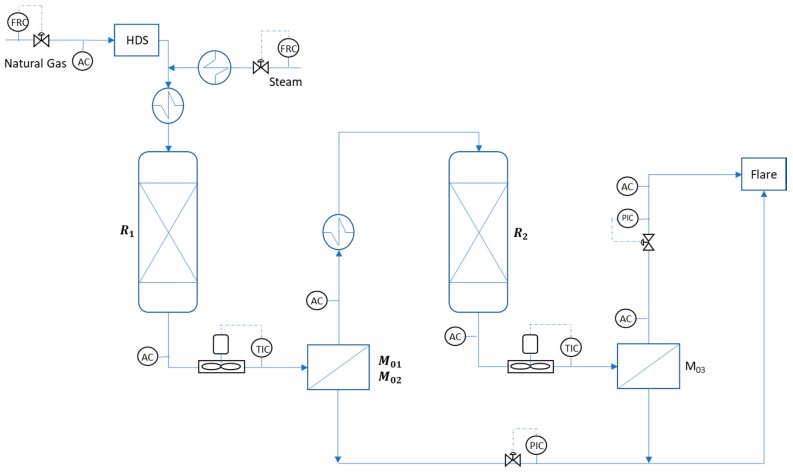
Simplified process scheme of the Pilot Plant, including two reactors and two membrane modules.

The Pilot Plant, realized by Tecnimont-KT Kinetics Technologies in Chieti (Italy), includes two reaction zones (R1, R2) and two separation zones, as depicted in the block diagram of Figure 2. The reaction zone is 15 m high, and consists of a radiant area (with burners and catalytic tubes) and a convective zone, where superheated steam is produced. The separation zone consists of two membrane separators—the first presenting two membranes (M-01 and M-02) working in parallel, and the second including a single module (M-03). The natural gas (NG) was provided by the NG network at 12 barg and desulfurized in the HDS unit. Steam was added to the feed and the mixture was preheated in the convective zone. The stream enters the first reformer where the reactions take place at 550–680 °C [16,17,18,19]. The effluent (syngas) is cooled in an air cooler to 450 °C, and routed to the first stage of separation. The depleted syngas (30–35% of H_2_ is removed) passes to the next reaction and separation zone. Permeates of both membranes are mixed and sent to the flare or to the cooling system in case of sweeping (water vapor condensation with cooling water in the closed cycle). Finally, the retentate stream is routed to the flare. The tests have been realized for 4 months to investigate the long-term stability of the structured catalyst and membrane modules, to carry out a comparison between structured catalyst and traditional Ni-based pellets, and to test the effectiveness of the RMM architecture integrated with the CPO reactor and to prove the improvement obtained by the RMM enhancement. Further details on the pilot plant can be found in the cited literature [16,17,18,19].

More than 70 tests, each running for around 10 h, were performed by varying the main operating conditions, such as temperature and pressure, as well as the steam-to-carbon ratio in the reforming section and the flowrate in the membrane modules. At the beginning of the test runs, the heating was realized by steam and nitrogen to reach a temperature above 300 °C (heating rate under 2.5–3 °C/min). Due to the endothermicity of the reaction, a slight temperature drop is observed after the feed introduction. This paper focused on the results related to the permeation zone, highlighted in Figure 2 and presented in Section 4. The main independent variable considered for the scaling-up correlations was the syngas flowrate (out of the reforming) that generates different mass transfer coefficients.

**Figure 3:**
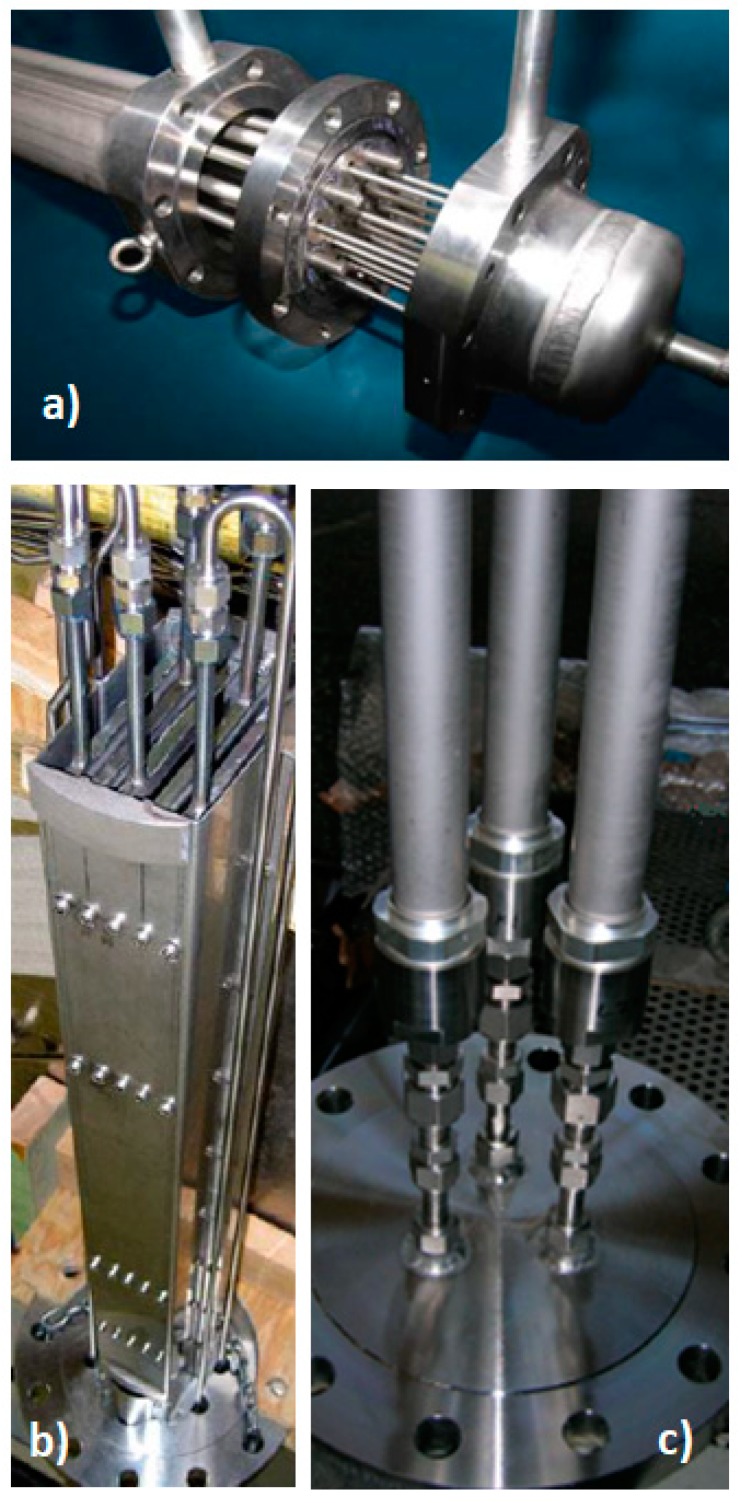
Photography of the membrane modules: (**a**) tubular M-01; (**b**) Flat Plates M-02; (**c**) tubular module M-03.

The main geometrical characteristics of the separation units are summarized in Table 1. The first membrane module, M-01, contained 13 tubular membranes of palladium supported on alumina (Figure 2a). The M-02 module (Figure 3b) consisted of five plate membranes, with each side consisting of a dense Pd-Ag layer deposited on the external surface of an α-alumina support (each side formed by two Pd-Ag panels welded in series). Both membranes can be used with sweeping gas. The last membrane module, M-03, used in the present work, was similar to M-01 but included three tubular membranes (Figure 3c).

The permeability value that accounts for the diffusion and solubility of hydrogen in palladium and palladium-silver alloys, was calculated for the different materials based on the theoretical and experimental works of Holleck [34] and Sarti et al. [28] according to the following general expression that diffusion is an energetically activated process and that Sievert’s constant represents the equilibrium reaction constant for hydrogen dissociation:

(1) $$\;P_{H_{2}}=\frac{1}{2}D_{0,H}\exp\left(\frac{\Delta S_{R}^{0}}{R}\right)\exp\left(-\frac{E_{D}+\Delta H_{R}^{0}}{RT}\right)=P_{H_{2}}^{0}\exp\left(-\frac{E_{a}}{RT}\right)\;$$

where $P_{H_{2}}^{0}$ is the permeability pre-exponential factor, and *E_a_* is the activation energy for hydrogen permeability which contains contributions from the activation energy for the diffusion of hydrogen atoms, the standard enthalpy of the surface dissociation reaction, as well as the entropy change of the dissociation reaction. These estimated values are reported in Table 1.

## 3. Mathematical Modelling

The present section describes the differential equations used to model the permeation phenomena and the procedure to estimate the mass transfer coefficient from the experimental data.

**Figure 4:**
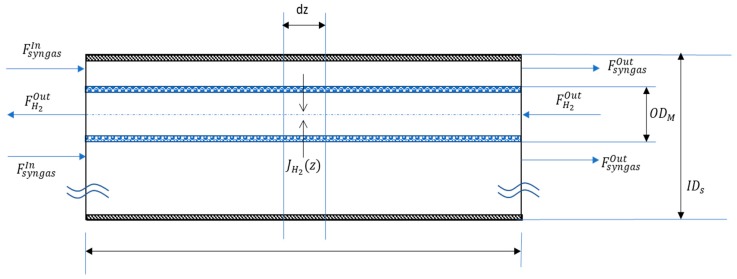
Reference scheme of the Material Exchanger for the mathematical model.

The main variables of the mathematical model are represented in Figure 4, which shows the longitudinal flux of the syngas (in the external tube) flowing in counter-current to the permeated flux of hydrogen and sweep gas (in the inner tube). The schematic representation is based on the experiments related to the shell and tube configuration (M-01 and M-03) with the H_2_ flux occurring from the outside to the inside of the tubes, where OD_t_ is the external diameter of the inner tube and ID_s_ is the diameter of the shell. The same configuration can be used for plate membranes (M-02) where the permeation channel is realized between two interspaces.

The membrane is simulated as an isotherm and isobar material exchanger (considering both permeance and molar fraction in the retentate side as constants) enabling the selective hydrogen permeation. Referring to the membrane’s infinitesimal volume of a length *dz*, the variation of the mass flow *F_j_* can be written as in Equations (2) and (3), respectively, for the permeate and the retentate side (with the minus sign if the flow is counter-current).

(2) $$\;\frac{d}{dz}F_{j}^{R}=-J_{j}\cdot 2\pi \left(r+\delta \right)\;$$

(3) $$\;\frac{d}{dz}F_{j}^{P}=\pm J_{j}\cdot 2\pi \left(r+\delta \right)\;$$

**Figure 5:**
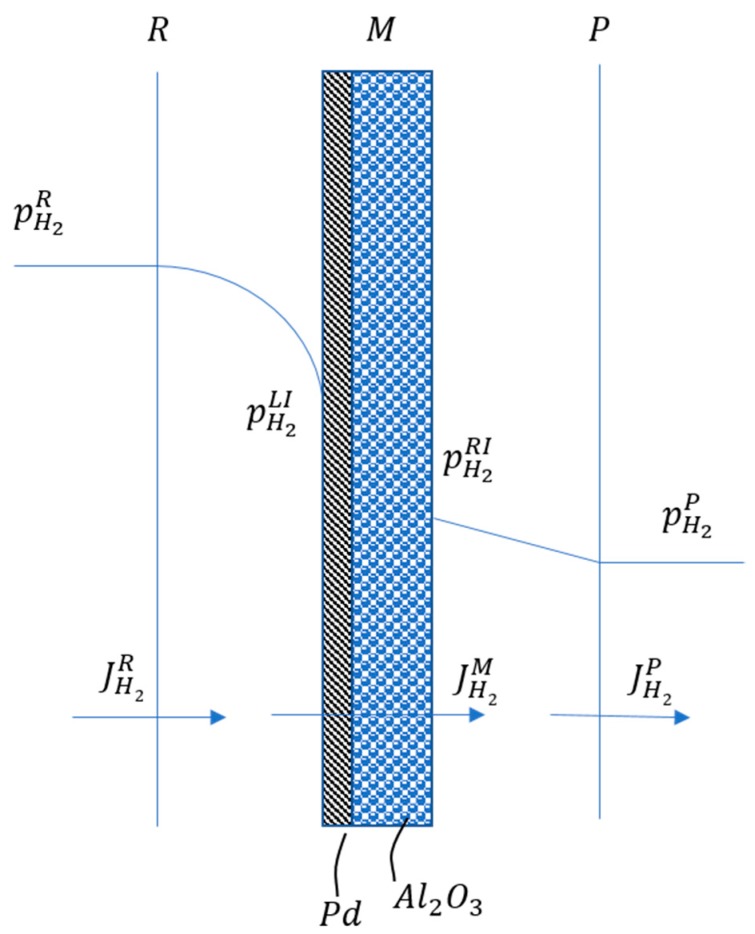
Schematic of the Film Theory considered for the flux characterization.

The Hydrogen flux *J_H_*_2_ through the membrane was calculated by adopting the schematization of the film theory represented in Figure 5. The mass transfer resistances considered in the diffusive process were concentrated from the bulk to the membrane wall on both the side of the membrane (retentate/permeate side), as well as through the palladium membrane (internal diffusion according to the Sievert-Fick law). In the absence of sweeping gas, the resistance in the permeate side is null and $p_{H2}^{RI}=p_{H2}^{P}$. The fluxes $J_{H2}^{R}$, $J_{H2}^{M}$, $J_{H2}^{P}$, respectively related to the driving force in the three abovementioned zones, are expressed by the Equations (4)–(6). For the experimental tests performed in absence of vapour sweeping, the resistance in the permeate side is neglected and the analysis focuses on the mass transport coefficient on the retentate side, explicated by Equation (7), that takes into account the concentration polarization phenomena with the mass transfer coefficient $F_{G}^{R*}$, depending on the concentration at the interface [35].

(4) $$\;J_{H2}^{R}=F_{G}^{R^{*}}\left(p_{H_{2}}^{R}-p_{H_{2}}^{LI}\right)\;$$

(5) $$\;J_{H2}^{M}=\frac{P_{H_{2}}}{\delta }\left(\sqrt{p_{H_{2}}^{LI}}-\sqrt{p_{H_{2}}^{RI}}\right)\;$$

(6) $$\;J_{H2}^{P}=F_{G}^{P^{*}}\left(p_{H_{2}}^{RI}-p_{H_{2}}^{P}\right)\;$$

(7) $$\;F_{G}^{R^{*}}=\frac{F_{G}^{R}}{{\left(p-p_{H_{2}}\right)}_{ML}}=\;F_{G}^{R}ln\left(\frac{1-y_{H_{2}}^{LI}}{1-y_{H_{2}}^{R}}\right)\;$$

A mathematical algorithm was implemented to estimate the mass transfer coefficients in the retentate side $\;\left(F_{G}^{R}\right)$ by regarding the observed experimental compositions, with partial pressure, as the unknown variable alongside the membrane. On the other hand, the membrane permeance was fixed on the basis of Equation (1) (calculated in agreement with the literature results [28,29,34]) by dividing the permeability $P_{H_{2}}$ by the membrane thickness δ, and thus does not represent a dependent variable of the mathematical model. The calculation procedure starts by assuming an initial value of *F_G_*. At each step, the algorithm calculates the molar fraction of hydrogen on the retentate side $\left(y_{H_{2}}^{LI}\right)$ by solving the nonlinear Equation (7) (by means of the Levenberg-Marquardt algorithm).

The Equations (9) and (10) show the linearization of the driving force of Sievert’s Law, here implemented to calculate the overall mass transfer coefficient *F_OG_*.

(8) $$\;\;J_{H2}^{R}=J_{H2}^{M}\;$$

(9) $$\;J_{H2}^{M}=\frac{P_{H_{2}}}{\delta }\frac{p_{H_{2}}^{LI}-p_{H_{2}}^{RI}}{\sqrt{p_{H_{2}}^{LI}}+\sqrt{p_{H_{2}}^{RI}}}\;$$

(10) $$\;\frac{1}{F_{OG}}=\frac{{\left(P-P_{H_{2}}\right)}_{ML}}{F_{G}}+\frac{\sqrt{P_{H_{2}}^{LI}}+\sqrt{P_{H_{2}}^{RI}}}{P_{H_{2}}/\delta }=\frac{1}{F_{G}^{*}}+\frac{1}{P_{H_{2}}^{*}}\;$$

The numerical integration was performed in Matlab by dividing the domain into 100 elements. The algorithm stopped when the difference between the flow of hydrogen on the permeate side (at the inlet of the membrane) was simulated by the mathematical model and the experimental value was minimized. In particular, an error of 1% between the calculated and experimental values was accepted.

Eventually, the estimated mass transfer coefficient $F_{G}^{R}$ could be correlated with the geometrical and operating conditions, keeping in mind that the experiments were realized with different membranes, at different temperature and flow rates by means of the non-dimensional numbers *Re*, *Sc,* and *Sh,* also depending on the physical properties of the mixture on the retentate side. The analysis of the competitive adsorption, well-characterized in the abovementioned papers [13,14,15], is kept outside of the model due to the low concentration of CO. The operation and geometrical parameters assumed to calculate these numbers are reported in Table 2, for the tubular (M-01 and M-02) and the plate (M-03) modules, respectively.

## 4. Results and Discussion

**Figure 6:**
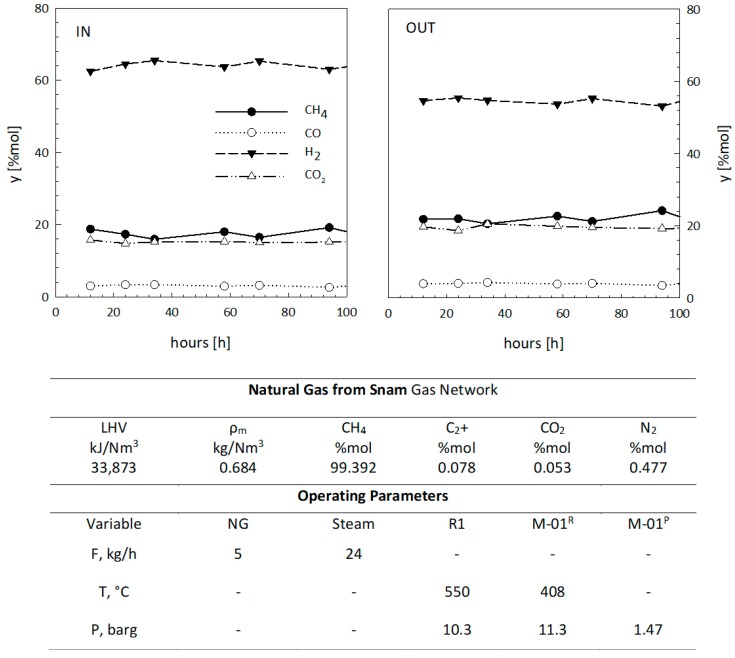
Complete report of a single set of experiments obtained at a fixed flow rate of natural gas (NG) and a single membrane arrangement (M-01). Averaged results from the other experiments are synthesized in Table 3, Table 4 and Table 5.

As described in Section 2, 70 test runs were selected for the purpose of this work. The experimental data obtained from a singular test (as a clarifying example) are reported in Figure 6. The inlet and outlet flowrates and temperatures were available as experimental data for the coefficient estimation.

The figure shows the gas concentrations both at the outlet of the first reactor (entrance to the membrane) and at the outlet of the membrane. The example is related to the first installed membrane module (M-01). The operating conditions are also reported, referring to the process diagram of Figure 2. The table also reports the characterization of the natural gas supplied to the overall RMM process.

At this point, to perform the mass transfer coefficient estimation, the experimental data were grouped in different levels of flowrate to find a possible correlation between F_G_ and the Reynolds number relative to the three tested membranes: five values for M-01, three values for M-02, and three values for M-03. These average experimental data (e.g., composition, hydrogen recovery, flow-rate, and permeability) are summarized in Table 3, Table 4 and Table 5.

**Figure 7:**
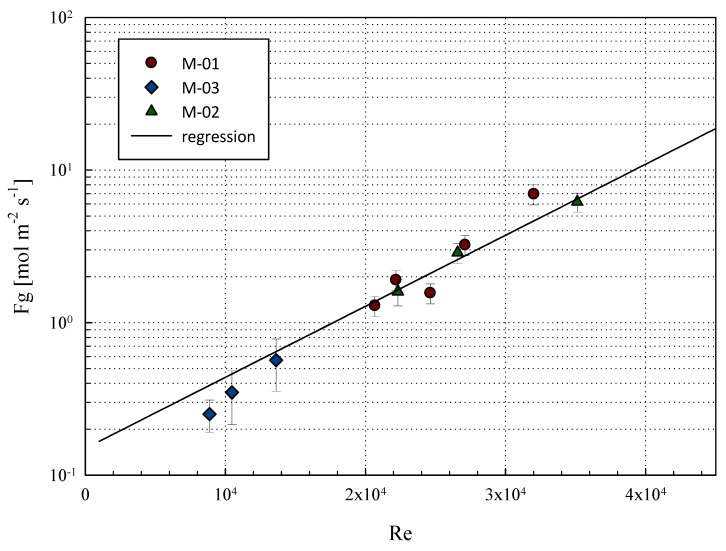
Mass transfer function of the Reynolds number.

By implementing the described procedure with the independent variables fixed at the values in Table 3, Table 4 and Table 5, the mass transfer coefficient *F_G_* was estimated. Figure 7 reports the F_G_ estimation results versus the Reynolds number characterizing each of the experiments. Each point represents the average value at each Reynolds number with error bars confined in the range of ±15%. The values globally follow a good linear correlation with the Reynolds number in the semi-log diagram, qualitatively confirming the observations made by Catalano et al. [28].

**Figure 8:**
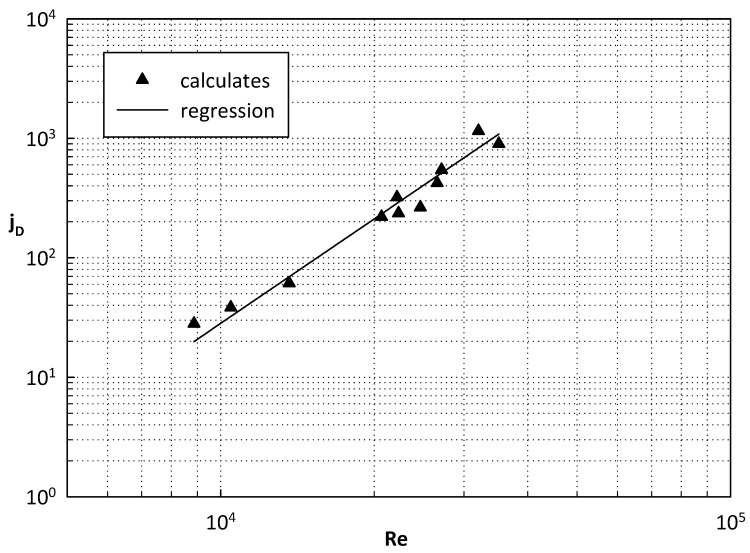
J_D_ vs. Re.

By adopting the approach of non-dimensional analysis, the mass transfer dimensionless group *j_D_* (Equation (11)) [35] can be found to correlate with the Reynolds number in Figure 8.

(11) $$\;j_{D}=Sh\cdot {\mathrm{Sc}}^{-1/3}\;$$

This representation produced a correlation equation (Equation (12)) which was valid in the range of tested conditions for the different geometries.

(12) $$\;j_{D}=2.172\times 10^{-10}\Delta Re^{2.79}\;$$

**Figure 9:**
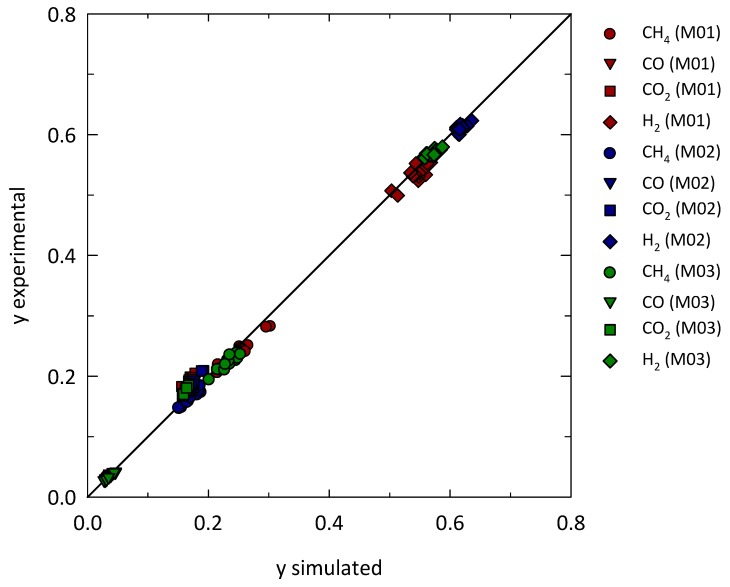
Parity plot of gas composition (molar fraction) for the whole experimental campaign.

Furthermore, to test again the reliability of the procedure, the obtained correlation (Equation (12)) was tested by reproducing the experimental results by implementing our model with a feedforward approach where, in this case, both the permeability and the F_G_ were known parameters and the latter was calculated though Equation (12). The modelling results, presented again in comparison with the observed ones, are in the form of calculated compositions at the exit of the membrane modules. These results are compared against the experimental values in the Parity diagram of Figure 9, finding an acceptable agreement. This last validation result confirms the goodness of the correlation obtained for the gas-side coefficient.

On this basis, referring to the film theory schematized in Figure 5, it is possible to calculate the relative weight of the mass transfer resistance *R* of the elementary steps (in-series) reported in Equations (13) and (14), respectively for the gas-phase in the retentate zone *R^cp^* (including the concentration polarization) and the one related to the permeation through the membrane *R^M^*.

(13) $$\;R^{cp}=\frac{{\left(P-P_{H_{2}}\right)}_{ML}}{F_{G}}\;$$

(14) $$\;R^{M}=\frac{\sqrt{P_{H_{2}}^{LI}}+\sqrt{P_{H_{2}}^{RI}}}{K_{H_{2}}}\;$$

**Figure 10:**
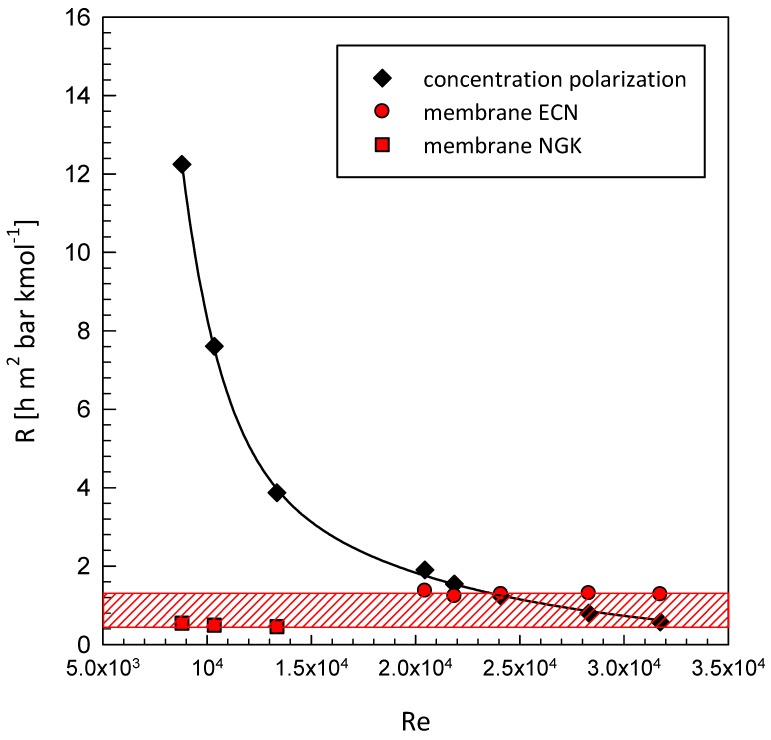
Effect of the Reynolds number on the mass transfer resistance in the gas phase and through the membrane.

Figure 10 shows the calculated resistance for the experimental conditions versus the Reynolds number. The resistance relative to the permeation step through the membrane is confined in a band (red) between the calculated values for the tubular (M-01,02) and plane (M-03) modules, having different thicknesses and different Pd-Ag ratios. The concentration polarization resistance *R^cp^* is an order of magnitude higher than the resistance *R^M^* due to the permeation in the membrane layer (according to the mechanisms postulated by Sieverts). It is worth noting that these data were obtained for membranes of different materials and different configurations. Therefore, in this work we enabled the calculation of the mass transfer coefficients in these peculiar “material exchangers” highlighting the two regimes of Figure 10, practically extending the work of Catalano et al. [27,28], being realized at very low numbers of Reynolds.

In the left side of the graph, the gas-side transport appears to be the limiting step; in the second, for Reynolds numbers greater than 25,000, the two resistances are comparable. As the number of Reynolds increases, the controlling step becomes the transport across the membrane, characterized by Sievert’s law.

## 5. Conclusions

The extensive use of hydrogen as a carrier would be a solution to the current conflict between economic expansion and pollution. It is also the only way to decarbonize the conversion processes of fossil fuels, waste, and biomass. In this paper, we gave new strength to the research on multistage reactors with intermediate hydrogen separation (so-called “RMM architecture”), focusing in particular on the “material exchanger” design, basically on the scale-up of these arrangements of metal membrane devices. The correlations now available in the literature focused on the estimation of the permeation direct from the Sievert Law (with or without changes of the exponent relating to partial pressures) resulting in a partial or inaccurate approximation of the phenomenology. Our approach, although still affected by simplifications, allows for a more accurate estimation of the transport coefficients in the membrane material exchangers for H_2_ separation, tested for different membrane layers and composition, as well as different operating conditions straddling two zones characterized by two different rate-limiting steps.

In this work, we reported the experimental tests obtained at the pilot plant, designed during the R&D Project, entitled “Pure hydrogen from natural gas reforming up to total conversion obtained by integrating chemical reaction and membrane separation”, and constructed by Tecnimont-KT Kinetics Technologies in Chieti (Italy). Many test runs have been collected and organized in relation to the flow rates and the membrane type.

A good correlation between our model and the experimental results validates the estimation of the mass transfer coefficient. Furthermore, it was possible to find reliable scaling-up correlations, including the whole set of data from different membrane configuration and operation conditions. The proposed correlation also allowed us to show that in the operating conditions, the mass transfer resistance due to concentration polarization can limit the hydrogen flux.

## Figures and Tables

**Table 1:** Characteristics of the experimental apparatus and operative conditions.

Geometrical Features	Membrane Modules		
	M-01	M-02	M-03
ID_S_, in	5	6	6
N_m_	13	5	3
OD_t_, mm	14	-	30
δ, μm	2.5	25	2.5
L, cm	69	30 × 2	45
A_ToT_, m^2^	0.4	0.6	0.13
T, °C	408–438	402–424	397–455
P^R^, bar	11–11.5	11.5	11
P^P^, bar	1.4–1.6	1.4	1.3
W, kg·h^−1^	29–46.4	29–46.4	29–46.4
F, kmol·h^−1^	1.9–3.1	1.9–3.1	1.9–3.1
$E_{a},$ kJ·mol^−1^	20.2	17.8	17.8
$P_{H_{2}}^{0}$, kmol·h^−1^·m^−2^·bar^−0.5^	1.69 × 10^−4^	2.67 × 10^−4^	2.67 × 10^−4^

**Table 2:** Parameters and dimensionless numbers in relation to the module characteristics.

Variables	Tubular Membrane (M-01, M-02)	Flat Plat Module (M-03)
*v*, m/s	$\frac{F}{3600\;\rho_{m}^{R}a_{s}}$	$\frac{F}{3600\;\rho_{m}^{R}a_{s}}$
$a_{s}$, m^2^	$\frac{\pi \left(ID_{s}^{2}-OD_{t}^{2}N_{m}\right)}{4}$	6 dw
$D_{eq}$, m	$4\frac{\left(P_{t}^{2}-\pi OD_{t}^{2}/4\right)}{\pi OD_{t}}$	$2\frac{dw}{d+w}$
Re	$\frac{\rho_{m}vD_{eq}}{\mu_{m}}$	
Sc	$\frac{\mu_{m}}{\rho_{m}D_{H_{2},m}}$	
Sh	$\frac{F_{G}^{R}D_{eq}}{c_{tot}D_{H_{2},m}}$	

**Table 3:** Experimental tests: Mean composition and operative conditions of M-01.

Parameter	Unit	1		2		3		4		5	
		IN	OUT	IN	OUT	IN	OUT	IN	OUT	IN	OUT
F	kmol·h^−1^	1.94	1.74	2.10	1.91	2.30	2.12	2.57	2.34	3.06	2.78
H_2_O	mol %	56	60	54	58	57	61	57	62	57	62
CO	mol %	1	2	1	1	1	1	1	1	1	1
CO_2_	mol %	6	7	6	7	6	7	6	7	6	7
CH_4_	mol %	8	9	11	13	8	9	9	10	9	10
H_2_	mol %	29	22	28	21	28	22	27	20	27	20
HRF	%	32		32		28		32		33	
$\bar{J}$	kmol·h^−1^·m^−2^	0.471		0.480		0.491		0.513		0.712	
$\bar{K_{H_{2}}}$	kmol·h^−1^·m^−2^·bar^0.5^	1.92		2.13		2.01		2.10		2.16	

**Table 4:** Experimental tests: Mean composition and operative conditions of M-02.

Parameter	Unit	1		2		3	
		IN	OUT	IN	OUT	IN	OUT
F	kmol·h^−1^	1.94	1.81	2.31	2.15	3.06	2.89
H_2_O	mol %	56	58	56	60	57	60
CO	mol %	1	2	1	1	1	1
CO_2_	mol %	6	7	6	7	6	6
CH_4_	mol %	8	9	9	9	9	10
H_2_	mol %	29	24	28	23	27	23
HRF	%	23		24		20	
$\bar{J}$	kmol·h^−1^·m^−2^	0.209		0.218		0.239	
$\bar{K_{H_{2}}}$	kmol·h^−1^·m^−2^·bar^0.5^	0.21		0.22		0.24	

**Table 5:** Experimental tests: Mean composition and operative conditions of M-03.

Parameter	Unit	1		2		3	
		IN	OUT	IN	OUT	IN	OUT
F	kmol·h^–1^	1.82	1.80	2.23	2.20	2.84	2.80
H_2_O	mol %	54	55	55	56	56	57
CO	mol %	2	2	2	2	1	1
CO_2_	mol %	8	8	8	8	8	8
CH_4_	mol %	7	7	7	7	7	7
H_2_	mol %	29	28	28	27	28	27
HRF	%	3		4		5	
$\bar{J}$	kmol·h^−1^·m^−2^	0.175		0.230		0.326	
$\bar{K_{H_{2}}}$	kmol·h^−1^·m^−2^·bar^0.5^	4.97		4.93		5.47	

## Nomenclature

$a_{s}$	shell area of membranes (m^2^);
$A_{tot}$	total membrane surface (m^2^);
$c_{tot}$	total concentration (kmol·m^−3^);
$D_{0,H}$	diffusion pre-exponential factor (m^2^·s^−1^);
$D_{H_{2},m}$	diffusivity of hydrogen in the mixture retentate side (m^2^·s^−1^);
$D_{eq}$	equivalent diameter of membranes (m);
d	channel depth (m);
$E_{a}$	activation energy for hydrogen permeation through metallic membranes (J·mol^−1^);
$E_{D}$	activation energy for the diffusion of hydrogen atoms (J·mol^−1^);
F	molar flow rate (kmol·h^−1^);
$F_{G}$	mass transfer coefficient (kmol·h^−1^·m^−2^);
$F_{OG}$	overall mass transfer coefficient (kmol·h^−1^·m^−2^);
HRF	hydrogen recovery factor;
$ID_{s}$	internal shell diameter (m);
$J_{H_{2}}$	hydrogen flux (kmol·h^−1^·m^−2^);
$\bar{J}$	average hydrogen flux (kmol·h^−1^·m^−2^);
$j_{D}$	mass transfer dimensional group;
$K_{H_{2}}$	hydrogen permeance (kmol·h^−1^·m^−1^·bar^−0.5^);
$\bar{K_{H_{2}}}$	average hydrogen permeance (kmol·h^−1^·m^−2^·bar^−0.5^);
L	membrane length (m);
LHV	lower heating value (kJ/Nm^3^);
$N_{G}$	natural gas (kg/h);
$N_{m}$	number of membranes;
$OD_{t}$	outside tube diameter (m);
$OD_{M}$	outside membrane diameter (m);
$P_{H_{2}}$	hydrogen permeability (kmol·h^−1^·m^−1^·bar^−0.5^);
$P_{H_{2}}^{0}$	permeability pre-exponential factor (kmol·h^−1^·m^−1^·bar^−0.5^);
$P_{t}$	tube pitch (m);
$p_{H_{2}}$	hydrogen partial pressure on the right/left interface (bar);
r	membrane internal radius (m);
Re	average Reynolds number;
v	average velocity of the mixture, retentate side (m·s^−1^);
Sc	average Schmidt number;
w	channel width (m);
$y_{H_{2}}$	hydrogen molar fraction on the retentate/lefth interface of i-esimo step;
*Apices and Subscripts*	
j	H_2_O, CO, CO_2_, CH_4_;
LI	relative to the left-interface in the film theory;
m	relative to the mixture;
cp	concentration polarization;
ML	logarithm mean;
P	relative to the permeate;
R	relative to the retentate-side;
$R^{*}$	relative to concentration polarization;
M	relative to membrane;
RI	relative to the right-interface in the film theory;
s	relative to the shell-side;
t	tube-side;
*Greekletter*	
δ	membrane thickness, (m);
$\mu_{m}$	viscosity of mixture, retentate side (Pa·s);
$\rho_{m}$	density of mixture, retentate side (kg·m^−3^);
$\Delta S_{R}^{0}$	standard enthalpy of the surface dissociation reaction (J·mol^−1^·K^−1^);
$\Delta H_{R}^{0}$	entropy change of the dissociation reaction (J·mol^−1^);
